# Addressing SARS-CoV-2 viroporins with antiarrhythmic drugs

**DOI:** 10.1093/europace/euae254

**Published:** 2024-10-16

**Authors:** Meye Bloothooft, Niels Voigt, Teun P de Boer

**Affiliations:** Department of Medical Physiology, Division of Heart & Lungs, University Medical Center Utrecht, Yalelaan 50, 3584 CM Utrecht, The Netherlands; Institute of Pharmacology and Toxicology, University Medical Center Göttingen, Göttingen, Germany; DZHK (German Centre for Cardiovascular Research), Partner Site Göttingen, Göttingen, Germany; Cluster of Excellence ‘Multiscale Bioimaging: from Molecular Machines to Networks of Excitable Cells’ (MBExC), University of Göttingen, Göttingen, Germany; Department of Medical Physiology, Division of Heart & Lungs, University Medical Center Utrecht, Yalelaan 50, 3584 CM Utrecht, The Netherlands

**This editorial refers to ‘SARS-CoV-2 ORF 3a-mediated currents are inhibited by antiarrhythmic drugs’ by Wiedmann *et al.*, https://doi.org/10.1093/europace/euae252.**

Infection with SARS-CoV-2 can have devastating effects, as became clear during the COVID-19 pandemic. The disease has been intensively studied, resulting in a wealth of information on many aspects of the acute disease and longer-term effects post-infection. Focusing on cardiac effects, several symptoms have been described in relation to SARS-CoV2 infection, ranging from myocarditis to cardiac arrhythmias.^1^ Typically, the aetiology of these arrhythmias is attributed to non-specific factors associated with COVID-19, such as fever, hypoxia, autonomic aberrations, and systemic inflammation.^2^ However, Wiedmann *et al*.^3^ propose a specific pathomechanism involving viral proteins that may function as ion channels, offering a more direct explanation for these disturbances.

It is well known that viruses can cause expression of ion channel-like proteins called viroporins. Viroporins play important roles in viral life cycle and participate in processes that are necessary for efficient viral entry, production, and release. As such, viroporins may represent valuable therapeutic targets for combating viral infections, potentially allowing for the repurposing of existing drugs to address these infections.^4^ Additionally, viroporins interfere with cellular functions, contributing to viral cytopathogenicity. Specifically, the disruption of intracellular ion homeostasis by viroporins can activate several harmful pathways, including apoptosis, the innate immune response, autophagy, and oxidative stress signalling.^5^ Since viroporins affect the conductance of the plasma membrane, effects on excitable cells are to be expected, which may be involved in the occurrence of cardiac arrhythmias associated with the SARS-CoV-2 infection. However, surprisingly little information can be found on the role of viroporins in cardiac arrhythmias. Although cardiac arrhythmias associated with viral infections, such as those caused by coxsackie B, adenovirus, and HIV, are thought to be due to alterations in intrinsic cardiac ion channels expressed in the host cardiomyocytes,^6^ to our knowledge, there are currently no studies specifically demonstrating that viroporins can alter cellular electrophysiology in cardiac myocytes. Furthermore, we were unable to identify any studies on other excitable cell types, such as neurons, where viroporins have been shown to cause abnormal excitations that contribute to the occurrence of seizures. All human coronaviruses express the open reading frame 3a (ORF3a) protein, and although the structure and function may differ between the various virus species,^7^ ORF3a is an important factor in SARS-CoV-2 pathogenicity; removing or blocking activity of the protein reduces severity of the infection and viral replication in host cells. This protein has also been suggested to act as a viroporin, and several studies have confirmed the ability of the protein to conduct ion current.^8,9^

In their study, Wiedmann *et al*.^3^ have asked the question whether infection of heart cells with SARS-CoV-2 results in expression of ORF3a, and whether ion currents conducted by ORF3a protein can be inhibited by antiarrhythmic drugs. Interestingly, upon infection with SARS-CoV-2, human-induced pluripotent stem cell-derived cardiomyocyte (hiPSC-CM) showed expression of ORF3a, but human atrial fibroblasts did not. When overexpressing ORF3a in *Xenopus laevis* oocytes, the authors detected outward potassium currents. Class I, II, or IV antiarrhythmic drugs had no or only minor effects, while class III drugs showed a blocking effect. These findings further support a role for ORF3a viroporins in cardiac arrhythmias associated with SARS-CoV-2. The outward potassium current conducted by ORF3a viroporins may increase the repolarization reserve of cardiac myocytes, an effect that can be reduced through administration of class III drugs such as amiodarone or dofetilide tested by Wiedmann *et al*.^3^ If the lack of expression of ORF3a in human atrial fibroblasts *in vitro* is representative of the situation *in vivo* during a SARS-CoV-2 infection, it could mean that the presence of viroporins changes the balance between electrically connected (via gap junctions) cardiomyocytes and fibroblasts. As such, differentially reducing the membrane resistance and excitability of cardiomyocytes but not fibroblasts is likely to affect tissue excitability. Furthermore, if there are regional differences in viral load in the heart, this may increase electrophysiological heterogeneity, which is known to be proarrhythmic.

Unfortunately, Wiedmann *et al*.^3^ have not reported on the effects of ORF3a on cardiac cellular electrophysiology, which could have been done by recording action potentials or ion currents in the infected hiPSC-CM. Measurements in iPSC-CM are not limited by artificial currents that may be induced in the *X. laevis* oocyte during RNA injection^10^ and that may lead to false positive current measurements. As such, and without additional experiments in cardiac myocytes, Wiedmann *et al*.^3^ extend the already available data on expression systems, where it cannot be ruled out that ion channel activity of viroporins is an artefact resulting from overexpression of the viroporins.^10^ Electron microscopy revealed that ORF3a displays a narrow constriction and the presence of a positively charged aqueous vestibule^11^ that would not favour cation permeation making activity as an ion channel unlikely.^12^ Furthermore, a recent study of ORF3a in a yeast-based model does support potassium conductance for ORF3a originating from SARS, but not SARS-CoV-2.^13^ Additionally, the use of iPSC-CM would have enabled authors to directly study effects of SARS-CoV-2 infection on cardiac cellular electrophysiology. These effects may be mediated by ORF3a viroporins but also by other more established viroporins such as the E protein. Finally, this system may have allowed the authors to identify modulation of host ion channels as a result of the SARS-CoV-2 infection.^5^

However, our speculations on potential effects of ORF3a viroporins on cardiac electrophysiology are exactly that, mere speculations. Biosafety requirements are not conducive to such experiments, but recording action potentials using voltage-sensitive dyes seems a feasible approach to study this further.^14^ Alternatively, overexpression of ORF3a alone in hiPSC-CM would be informative and much easier.

Taken together, the effects of viral proteins on cellular electrophysiology represent an emerging field that has long been overlooked by virology and biophysical researchers. It certainly deserves greater attention, and Wiedmann *et al*.^3^ should be commended for drawing attention to this important research area.
